# A Rare Cause of Sleep-Disordered Breathing: ROHHAD Syndrome

**DOI:** 10.3389/fped.2020.573227

**Published:** 2020-11-20

**Authors:** Gizem Özcan, Elif Özsu, Zeynep Şiklar, Nazan Çobanoğlu

**Affiliations:** ^1^Department of Pediatric Pulmonology, Faculty of Medicine, Ankara University, Ankara, Turkey; ^2^Department of Pediatric Endocrinology, Faculty of Medicine, Ankara University, Ankara, Turkey

**Keywords:** childhood, hypoventilation, hypothalamic dysfunction, ROHHAD syndrome, sleep disordered breathing

## Abstract

Rapid-onset obesity with hypoventilation, hypothalamic dysfunction, and autonomic dysregulation (ROHHAD) syndrome; is a rare but crucial disorder. Sleep-disordered breathing can occur at the beginning or after of obesity. A disease-specific test for diagnosis is not yet available. Neural crest tumors (ganglioneuroma, ganglioneuroblastoma) have been reported in 40% of patients. In our study, three patients diagnosed as having ROHHAD syndrome are presented from our hospital. In the evaluation of the hypothalamic functions of the patients, one of them had growth hormone deficiency and hyperprolactinemia; recurrent hypernatremia reflecting irregular water balance was detected in another. One of the patients had abnormal pupil reflex and heart rate irregularity while another had excessive sweating as autonomic dysfunction. One of the patients was diagnosed with paravertebral ganglioma accompanying ROHHAD syndrome. Non-invasive ventilation treatment was started in all patients because there was a sleep-disorder breathing clinic diagnosis. ROHHAD syndrome deserves a multidisciplinary team approach as it can affect more than one organ system. In these patients, should be sleep-disorder breathing determined early and appropriate treatment should be initiated immediately to reduce morbidity and mortality.

## Introduction

Hypoventilation means carbon dioxide retention in arterial blood as a result of deficient gas exchange, and a deficient central nervous system function is the main problem in central hypoventilation. Congenital or acquired conditions may lead to central hypoventilation. Unless these conditions are identified, central hypoventilation can cause morbidity and mortality (1). Rapid-onset obesity with hypoventilation, hypothalamic dysfunction, and autonomic dysregulation (ROHHAD) syndrome, which is one of the causes of congenital central hypoventilation, was first described in 1965 (2). However, since the detection that mutations in the PHOX2B gene lead to congenital hypoventilation syndrome (CCHS) (3), ROHHAD syndrome has been distinguished from CCHS as a separate condition (4, 5).

Presentation with hyperphagia and remarkable weight gain ages between 2 and 9 years is typical in patients with ROHHAD syndrome, although they are healthy prior to the start of the aforementioned symptoms (4, 6). Later, patients may have clinics showing impaired hypothalamic functions such as central hypothyroidism, growth hormone (GH) deficiency, hyperprolactinemia, precocious or delayed puberty, hyponatremia or hypernatremia, and adrenocorticotropic hormone (ACTH) deficiency (7). Increased sweating, thermal disturbances, gastrointestinal tract dysmotility, cold hands, and feet, dilated pupils with abnormal reaction to light, decreased sensitivity to pain, and bradycardia are the symptoms of autonomic dysregulation. Delay or regression of psychomotor development, depression, emotional lability, panic and anxiety attacks, psychotic symptoms, and seizures can be seen as behavioral disorders (4–8). Early-onset obstructive sleep apnea and then central alveolar hypoventilation may develop overtime in these patients (9). In ROHHAD syndrome, ganglioneuromas, and ganglioneuroblastomas, which are tumors of neural crest origin, have been found in 33–40% of cases (6, 7, 10).

Clinical criteria are *sine qua non* for the diagnosis of ROHHAD syndrome. Basic cardiopulmonary, central nervous system, and neuromuscular evaluations are also essential both for differential diagnosis and search out complications (11). Management of those complications and early diagnosis of ROHHAD syndrome have great importance for successful management.

Special attention should be paid to the set of sleep-disordered breathing (SDB) due to the need for respiratory support. In order to highlight this condition and promote optimal management of future patients, we present three patients for whom we commence respiratory support at the time of diagnosis.

## Case Reports

### Case 1

A 7.5-year-old female patient with the symptoms sleeping, difficult awakening, and decline in school performance 10 months before admission to our hospital was evaluated by pediatric neurology and psychiatry. She continued to be symptomatic despite treatment with methylphenidate, which was started with a diagnosis of narcolepsy. Six months after the onset of symptoms, she had a 6-day admission in pediatric intensive care unit (PICU), following a cardiac arrest during feeding, and was intubated and ventilated for 24 h. Two months later, she had a second cardiac arrest and was readmitted. The patient was extubated after 3 weeks and non-invasive ventilation (NIV) was commenced due to continued CO_2_ retention. She had a further assessment in our PICU and the medical history of the patient revealed that she was born at term with adequate birth weight and length. There was no consanguinity between the parents and she had two healthy siblings (ages 9 and 6 years). The parents reported that she was growing and developing normally initially but began to gain weight rapidly at age 5 years. Later, at the age of six, hypersomnia began. Her body weight was 35 kg (+2.05 SD), and height was 119 cm (−0.95 SD) with a body mass index (BMI) 24.8 kg/m^2^ (+2.61 SD). Dilated pupils with an abnormal reaction to light were detected in an eye examination. There were no other abnormalities on physical examination.

A series of laboratory analyses revealed normal results for biochemical studies of blood and urine except respiratory acidosis in the blood gas analysis. Her hormonal evaluation revealed the following results: free T4, 9.2 pmol/L; thyroid stimulating hormone (TSH), 8.20 μiu/mL; adrenocorticotropic hormone (ACTH), 57.94 pg/mL; cortisol, 18.77 μg/dL; luteinizing hormone (LH), <0.1 mIU/mL; estradiol, 29.21 pg/mL; prolactin (PRL), 42 ng/mL (NR: 4.7–23.3 ng/mL). Insulin like growth factor-1 (IGF-1) was 52 ng/mL (-2.8 SD) and IGF binding protein 3 (IGFBP3) 3,460 ng/mL (−3.7 SD). Cranial magnetic resonance imaging (MRI) showed cortical atrophy with ventricular enlargement; however, a pituitary MRI was normal. Electroencephalography (EEG) was normal. There was no abnormality in echocardiography and 24 h rhythm Holter monitoring. A molecular study of the PHOX2B gene revealed no abnormalities. Upon the detection of multiple atelectasis on thorax computed tomography (CT), the patient underwent flexible fiberoptic bronchoscopy. Her antibiotherapy was regulated and thoracic postural drainage was performed regularly. Polysomnography (PSG) could not be performed for the diagnosis of SDB because PSG was not available at our hospital. However, during the follow-up, it was observed that her CO_2_ retention in her arterial blood gas analysis developed when there was no NIV support. A home non-invasive mask ventilation program during the night was scheduled for the patient. Bi-level positive airway pressure (BiPAP) in spontaneous/timed (S/T) mode with inspiratory positive airway pressure (IPAP) of 14 cm of water pressure (CWP), expiratory positive airway pressure (EPAP) of seven CWP, and back up respiratory rate of 20/min was started. She was compliant with the BiPAP therapy during 3 years of follow-up. Despite regression of CO_2_ retention after NIV treatment, daytime sleepiness persisted, and a pediatric neurology specialist prescribed methylphenidate first, and then modafinil, and a partial response was received. Due to her depression, she has continued psychiatry outpatient visit. GH treatment and PSG monitoring for guiding have been planned.

### Case 2

Case 2 was a 5-year-old male patient, who was born at term and weighed 2,900 g at birth. He started overeating at age of 4 years. He gained 22 kg in a year despite being on diet. When the parents were questioned, it was learned that he had started snoring, he was having breath holding episodes and waking up with heavy sighs. His body weight was 35 kg (+5.07 SDS), and height was 119 cm (+0.17 SDS) with a body mass index (BMI) 33.1 kg/m^2^ (+4.97 SD). The patient had “buffalo hump” but no other pathological findings on physical examination.

His kidney and liver function tests and complete blood count were normal. His hormonal evaluation revealed the following results: free T4, 19.45 pmol/L; TSH, 3.07 μ1u/mL; ACTH, 33.4 pg/mL; cortisol, 15.08 μg/dL; LH, <0.1 mIU/mL; FSH, <0.3 mIU/mL; testosterone, <0.1 ng/dL; prolactin (PRL), 45.25 ng/mL. IGF-1 was 8.0 ng/mL (−3.6 SDS) and IGFBP3 2,000 ng/mL (−0.33 SDS). Cranial and pituitary MRI were normal, concentric hypertrophy of the left ventricle was detected on his echocardiography. He had grade 2 hepatosteatosis. PSG was performed to the patient and apnea hypopnea index (AHI) was found as 48.3 (obstructive apnea 22%, central apnea 78%). BPAP in ST mode (with IPAP: 12 CWP, EPAP: 4 CWP, back up respiratory rate of 20/min) was begun. Thorax CT images were obtained was taken from the hospital he had presented to previously and there was a paravertebral mass appearance. Thorax MRI was performed in our hospital because neural crest tumors have been reported with ROHHAD syndrome. There was a paravertebral mass on T7–9 vertebra level so tumor excision was performed with video-assisted thoracoscopic surgery. The histopathologic diagnosis was ganglioneuroma. There were no intra-operational or post-operational complications. He has been on follow-up for 2 years.

### Case 3

An 8-year-old girl attended the hospital reporting cyanosis on the lips and unconsciousness after exertion. The patient, who had rales on auscultation, was admitted with the diagnosis of pneumonia, and antibiotic therapy was commenced. She began on NIV for hypercarbia and this was followed by intubation and mechanical ventilation. Hyponatremia and liver dysfunction were present in her laboratory tests. Pulmonary hypertension was detected in her echocardiography. She was admitted to our PICU after 4 days of hospitalization where a further assessment was undertaken.

She was born at term with adequate birth weight and length. There was no consanguinity between parents and she had three healthy siblings. She had rapid weight gain with uncontrollable eating, starting at 5 years of age. For the past 1 month, her mother noticed that she was holding her breath asleep. She had excessive sweating. During hospitalization, we noticed that she did not complain or cry when she underwent needle phlebotomy. Her weight was 50 kg (+3.52 SDS), and her height was 135 cm (+1.43 SDS) with a body mass index (BMI) 27.4 kg/m^2^ (+3.44 SDS).

Laboratory evaluation revealed sodium (Na), 155 mmol/L [normal range (NR): 135–145 mmol/L]; aspartate transaminase, 315 U/L (NR: 8–45 U/L); alanine aminotransferase, 798 U/L (NR: 7–55 U/L); and urine density, 1,021. The remaining biochemical studies of blood were normal. As the patient had no polydipsia, hypernatremia was assumed to be due to insufficient intake. The hypernatremia was corrected (Na: 141 mmol/L) with intravenous fluid replacement therapy. No abnormal value was detected in her hormonal tests and pituitary MRI was normal. Diffusion restriction in white matter was detected in cranial MRI; therefore, cranial MRI spectroscopy was performed but no pathology was detected. Thoracic postural drainage was initiated in the patient due to widespread atelectasis on thorax CT. The patient was extubated after 24 h in the PICU and NIV treatment was initiated. She was discharged from the PICU 3 days later. The patient's CO_2_ retention continued during sleep when off NIV support, and she was therefore discharged using BPAP in ST mode (with IPAP: 15 CWP, EPAP: 7 CWP, back up respiratory rate of 15/min) during sleep. In the outpatient follow-up 1 month later, the patient was again diagnosed as having adipsic hypernatremia, which was treated with oral fluid support. She was referred to pediatric psychiatrist because the family expressed emotional lability in the patient.

Clinical, laboratory, and imaging findings at the time of diagnosis are presented also in Tables 1, 2.

**Table 1:** Clinical findings.

**Clinical Findings**	**Case1**	**Case 2**	**Case 3**
Age of diagnosis	7.5 years	4 years	8 years
Age at which rapid weight gain began	5 years	3 years	5 years
Follow-up period	37 months	24 months	4 months
Hypothalamic dysfunction	Hyperprolactinemia	Hyperprolactinemia	Hypernatremia
	Deficiency of growth hormone		
Sleep disordered breathing	CO_2_ retention in arterial blood gas analyses during sleeping	Polysomnography	CO_2_ retention in arterial blood gas analyses during sleeping
Autonomic dysfunctions	Abnormal pupil reflex	–	Excessive sweating
	Heart rate irregularity		Decreased pain sensitivity
Neural crest tumor	–	Ganglioneuroma	-
Mood disorder	+	–	+

**Table 2:** Laboratory and imaging findings.

	**Case 1**	**Case 2**	**Case 3**
**Endocrinological parameters**			
Prolactin			
IGF-1	**42 ng/mL**	**45.25 ng/mL**	18.35 ng/mL
IGFBP-3	**52 (−2.8 SDS)**	**8.0 ng/mL (−3.6 SDS)**	
TSH	**3,460 (−3.7 SDS)**	2,000 ng/mL (−0.33 SDS)	
fT4	8.20 μ1u/mL	3.07 μ1u/mL	0.73 μ1u/mL
ACTH	9.2 pmol/L	19.45 pmol/L	13.54 pmol/L
Plasma Cortizol	57.94 pg/mL	33.4 pg/mL	9.54 pg/mL
LH	18.77 μg/dL	15.08 μg/dL	32.06 μg/dL
FSH	<0.1 mIU/mL	<0.1 mIU/mL	<0.2 mIU/mL
DHEAS	1.03 mIU/mL	<0.3 mIU/mL	0.57 mIU/mL
Testosterone			
17-OH Progesterone		<0.1 ng/dL	
Estradiol	0.75 ng/mL 29.21 pg/mL		
Pituitary magnetic resonance imaging (MRI)	Normal	Normal	Normal
Cranial MRI	Cerebral atrophy, ventricular enlargement	Normal	White matter posterior diffusion restriction, Focal atrophy compatible with old ischemia in left-back parietal (MR spectroscopy: Normal)
Echocardiography	Normal (Holter: Normal)	Concentric hypertrophy of the left ventricle	Normal
PHOX2B mutation	PHOX2B (–)	Unknown	Unknown
SNRPN (15q11–q13) mutation	SNRPN Unknown		

*Parameters in bold are pathological*.

## Discussion

Until the onset of symptoms, children with ROHHAD syndrome show normal growth and development. Symptoms can develop over the years (4). Rapid-onset obesity is the most common initial symptom, as in our cases. Other conditions accompanied by obesity should be checked. Children with exogenous obesity have higher IGF-1 levels and these patients are often tall. By contrast, patients with ROHHAD syndrome have both low serum IGF-1 levels and insufficient response to GH stimulatory tests (12).

The endocrine features of hypothalamic dysfunction are highly variable in ROHHAD syndrome. Hyperprolactinemia, adrenal insufficiency, central hypothyroidism, GH deficiency and pubertal development abnormalities may also occur (4, 7). Hyperprolactinemia was present in two of our patients (Case 1 and 2), but no treatment was started due to its mild level. Hypothalamic dysfunction in children may be related to congenital malformations, trauma, or hypothalamic tumors. Therefore, radiological imaging of the central nervous system has great importance. The pituitary MRIs of our patients were normal. Two of our patients had cerebral atrophy in cranial MRI, and this finding was reported in previous case reports (8, 13). Thirst and antidiuretic hormone secretion abnormalities related to hypernatremia or hyponatremia are also frequently reported symptoms of hypothalamic dysfunction. In Case 3, the presence of hypernatremia was considered irrelevant to DI due to the absence of polyuria and polydipsia. As both urine osmolality and density were normal, she was accepted as having adipsic hypernatremia. The presence of abnormal pupil reflex and heart rate irregularity in one patient and excessive sweating and decreased pain sensitivity in another supported autonomic dysfunction. In patients with ROHHAD syndrome, psychiatric symptoms, and mood changes (hypersomnia/ insomnia, severe anxiety, auditory hallucinations, and disabling fears) have been defined previously (4, 6). Therefore, follow-up of patients is also recommended in this regard (14). Two of our patients (Case 1 and 3) were referred to pediatric psychiatrist due to mood changes during follow-up.

Central hypoventilation leads to progressive inadequate ventilation, combination of central hypoventilation with dysautonomia, carries a high risk of sudden death (11). Although the frequency of SDB is not fully known in patients with ROHHAD syndrome, all patients evaluate this aspect as part of the disease. Except for central hypoventilation, which is required for diagnosis, obstructive sleep apnea has also been frequently reported at disease onset. Reppucci et al. (9) emphasized that all patients with a pre-diagnosis of ROHHAD syndrome should undergo PSG in diagnosis and follow-up, but it should be emphasized that the diagnosis should not be excluded only by detecting obstructive sleep apnea and that nocturnal hypoventilation might develop during follow-up of these patients. Nevertheless, pathophysiology of the disease remains to be determined. Carroll et al. (15) showed that the responses of patients with ROHHAD syndrome to their body against hypoxia and hypercarbia were in many ways the same as healthy young control groups' responses. If diminished inspiratory drive and tidal volumes are combined with a lack of behavioral perception of asphyxia, these patients are thought to become more susceptible to hypoxia and hypercarbia. Although the gold standard for SDB is PSG, we could not perform PSG for the first and third patients because the number of sleep laboratories in our country where PSG can be performed in children is very limited. There are many referrals to the sleep laboratories and it takes a long time for appointments. For this reason, we made the diagnosis of SDB through clinical findings and arterial blood gas analysis and we performed NIV treatment in order not to lose time until PSG could be performed.

A mutation in PHOX2B gene encoding transcription factor leads to congenital central hypoventilation syndrome (CCHS), and keeping this syndrome in mind in differential diagnosis of patients with ROHHAD syndrome and SDB is important (1, 3). In most of the cases, CCHS manifests in the neonatal period (5). All of our patients developed obesity after 2 years of age, and in all of them autonomic and hypothalamic dysfunction occurred later in the course of the disease. It has been shown that 50% of the patients need respiratory support that ranges from nocturnal non-invasive ventilation to continuous mechanical ventilation in the widest two case series (4, 6).

In conclusion, ROHHAD syndrome can be presented with different clinical scenarios, as was the case in our patients. It is important to consider ROHHAD syndrome when assessing a patient with rapid-onset obesity. Another important point we want to highlight is that respiratory problems are important causes of morbidity in these patients. All patients with a pre-diagnosis of ROHHAD syndrome should be evaluated in terms of SDB and if necessary, NIV treatment should be taken.

## Data Availability Statement

The original contributions presented in the study are included in the article/supplementary material, further inquiries can be directed to the corresponding author/s.

## Ethics Statement

Written informed consent for the publication of this case report was obtained from patient's parents.

## Author Contributions

GÖ and EÖ performed the literature review and wrote the manuscript. ZŞ and NÇ critically reviewed the manuscript. All authors contributed to the article and approved the submitted version.

## Conflict of Interest

The authors declare that the research was conducted in the absence of any commercial or financial relationships that could be construed as a potential conflict of interest.

## References

[1] Cielo C Marcus CL Central hypoventilation syndromes. Sleep Med Clin. (2014) 9:105–18. 10.1016/j.jsmc.2013.10.005 24678286 PMC3963184

[2] Fishman L Samson J Sperling P. Primary alveolar hypoventilation syndrome (Ondine's Curse). Am J Dis Child. (1965) 110:155–61. 10.1001/archpedi.1965.02090030165011 14320765

[3] Amiel J Laudier B Attié-Bitach T Trang H De Pontual L Gener B Polyalanine expansion and frameshift mutations of the paired-like homeobox gene PHOX2B in congenital central hypoventilation syndrome. Nat Genet. (2003) 33:459–61. 10.1038/ng1130 12640453

[4] Ize-Ludlow D Gray JA Sperling MA Berry-Kravis EM Milunsky JM Farooqi IS Rapid-onset obesity with hypothalamic dysfunction, hypoventilation, and autonomic dysregulation presenting in childhood. Pediatrics. (2007) 120:e179–88. 10.1542/peds.2006-3324 17606542

[5] Katz ES McGrath S Marcus CL. Late-onset central hypoventilation with hypothalamic dysfunction: a distinct clinical syndrome. Pediatr Pulmonol. (2000) 29:62–8. 10.1002/(SICI)1099-0496(200001)29:1<62::AID-PPUL10>3.0.CO;2-M 10613788

[6] De Pontual L Trochet D Caillat-Zucman S Abou Shenab OA Bougneres P Crow Y. Delineation of late onset hypoventilation associated with hypothalamic dysfunction syndrome. Pediatr Res. (2008) 64:689–94. 10.1203/PDR.0b013e318187dd0e 18670370

[7] Bougnères P Pantalone L Linglart A Rothenbühler A Le Stunff C. Endocrine manifestations of the rapid-onset obesity with hypoventilation, hypothalamic, autonomic dysregulation, and neural tumor syndrome in childhood. J Clin Endocrinol Metab. (2008) 93:3971–80. 10.1210/jc.2008-0238 18628522

[8] Chew HB Ngu LH Keng WT New disease Rapid-onset obesity with hypothalamic dysfunction, hypoventilation and autonomic dysregulation (ROHHAD): a case with additional features and review of the literature. BMJ Case Rep. (2011) 2011:1–6. 10.1136/bcr.02.2010.2706 PMC3062047 22715259

[9] Reppucci D Hamilton J Yeh EA Katz S Al-Saleh S Narang I. ROHHAD syndrome and evolution of sleep disordered breathing. Orphanet J Rare Dis. (2016) 11:1–8. 10.1186/s13023-016-0484-1 27473663 PMC4967322

[10] Lee JM Shin J Kim S Gee HY Lee JS Cha DH. Rapid-onset obesity with hypoventilation, hypothalamic, autonomic dysregulation, and neuroendocrine tumors (ROHHADNET) syndrome: a systematic review. Biomed Res Int. (2018) 2018:1–17. 10.1155/2018/1250721 30584530 PMC6280256

[11] Patwari PP Wolfe LF. Rapid-onset obesity with hypothalamic dysfunction, hypoventilation, and autonomic dysregulation: review and update. Curr Opin Pediatr. (2014) 26:487–92. 10.1097/MOP.0000000000000118 24914877

[12] Abaci A Catli G Bayram E Koroglu T Olgun HN Mutafoglu K. A case of rapid-onset obesity with hypothalamic dysfunction, hypoventilation, autonomic dysregulation, and neural crest tumor: Rohhadnet syndrome. Endocr Pract. (2013) 19:12–6. 10.4158/EP12140.CR 23186956

[13] Kocaay P Siklar Z Çamtosun E Kendirli T Berberoglu M. Case report difficulties in obesity. J Clin Res Pediatr Endocrinol. (2014) 6:254–7. 10.4274/jcrpe.1432 25541898 PMC4293662

[14] Weese-mayer DE Bs CMR Ize-ludlow D. Commentary: rapid-onset obesity with hypothalamic circulation. J Can Acad Child Adolesc Psychiatry. (2013) 22:238–9. 23970914 PMC3749899

[15] Carroll MS Patwari PP Kenny AS Brogadir CD Stewart TM Weese-Mayer DE. Rapid-onset obesity with hypothalamic dysfunction, hypoventilation, and autonomic dysregulation (ROHHAD): Response to ventilatory challenges. Pediatr Pulmonol. (2015) 50:1336–45. 10.1002/ppul.23164 25776886

